# Selective cytotoxicity of zinc peroxide and tetrapodal zinc oxide micro-nanoparticles against breast cancer cells: synthesis, characterization, and therapeutic potential

**DOI:** 10.1007/s10856-026-07091-6

**Published:** 2026-06-24

**Authors:** Tehseen Riaz, Marcus Lettau, Mady Elbahri, Marie Elis, Nicholas Dunne, Tanya J. Levingstone, Jörg P. Weimer, Marion van Mackelenbergh, Norbert Arnold, Nicolai Maass, Dirk Bauerschlag, Fabian Schütt, Rainer Adelung, Nina Hedemann

**Affiliations:** 1 Chair for Functional Nanomaterials, Institute for Materials Science, Kiel University, Kiel, Germany; 2 School of Mechanical and Manufacturing Engineering, Dublin City University, Dublin, Ireland; 3 Institute of Immunology, Universitätsklinikum Schleswig-Holstein (UKSH), Kiel, Germany; 4 Department of Chemistry and Materials Science, Aalto University, Espoo, Finland; 5 Chair for Synthesis and Real Structure, Institute for Materials Science, Kiel University, Kiel, Germany; 6 Centre for Medical Engineering Research, Dublin City University, Dublin, Ireland; 7 Advanced Manufacturing Research Centre (I-Form), School of Mechanical and Manufacturing Engineering, Dublin City University, Dublin, Ireland; 8 School of Pharmacy, Queen’s University Belfast, Belfast, UK; 9 Biodesign Europe, Dublin City University, Dublin, Ireland; 10 Advanced Processing Technology Research Centre, Dublin City University, Dublin, Ireland; 11 Research Centre for Medical Devices (CÚRAM), Biomedical Sciences, University of Galway, Galway, Ireland; 12 Advanced Materials and Bioengineering Research Centre (AMBER), Trinity College Dublin, Dublin, Ireland; 13 Department of Gynecology and Obstetrics, Universitätsklinikum Schleswig-Holstein (UKSH), Kiel, Germany; 14 Clinic and Polyclinic for Gynaecology and Reproductive Medicine, University Hospital Jena, Jena, Germany; 15 Unit for 3D-Patient Avatars and Personalized Medicine, Department of Gynecology and Obstetrics, Christian-Albrechts-University Kiel & University Hospital Schleswig-Holstein, Kiel, Germany

## Abstract

Breast cancer remains a global challenge, with rising incidence rates. While treatment advancements have improved outcomes, systemic administration of cytostatic agents continues to cause severe adverse effects, including myelosuppression, heart failure, and infertility, due to non-specific biodistribution. Nanoparticle-based drug delivery systems have been explored as an alternative approach to improve therapeutics delivery and reduce non-specific effects. Zinc-based nanoparticles demonstrate ability to induce cytotoxic responses in cancer cells in vitro, making them suitable systems for probing cell-type-specific effects. In this study, we evaluated the in vitro cytotoxic effects of spherical zinc peroxide (ZnO₂) nanoparticles (20–80 nm and 50–300 nm), commercial ZnO nanoparticles, and tetrapodal ZnO (T-ZnO) microparticles in MCF-7 breast cancer cells and RMF-EG fibroblasts. ZnO₂ nanoparticles demonstrated dose-dependent cytotoxicity (1 µg/mL–10 mg/mL), inducing a significantly higher death rate of cancer cells than normal fibroblasts, which retained >75% viability at comparable doses. Flow cytometry investigations revealed that ZnO₂ nanoparticles exhibited preferential cellular uptake in MCF-7 cells as compared to fibroblasts. Overall, these findings indicate different cytotoxic responses of ZnO-based micro and nanoparticles between MCF-7 cells and RMF-EG fibroblasts under in vitro conditions. Further studies are required to validate these observations in more complex biological systems and to clarify the underlying mechanisms.

**Graphical Abstract:**

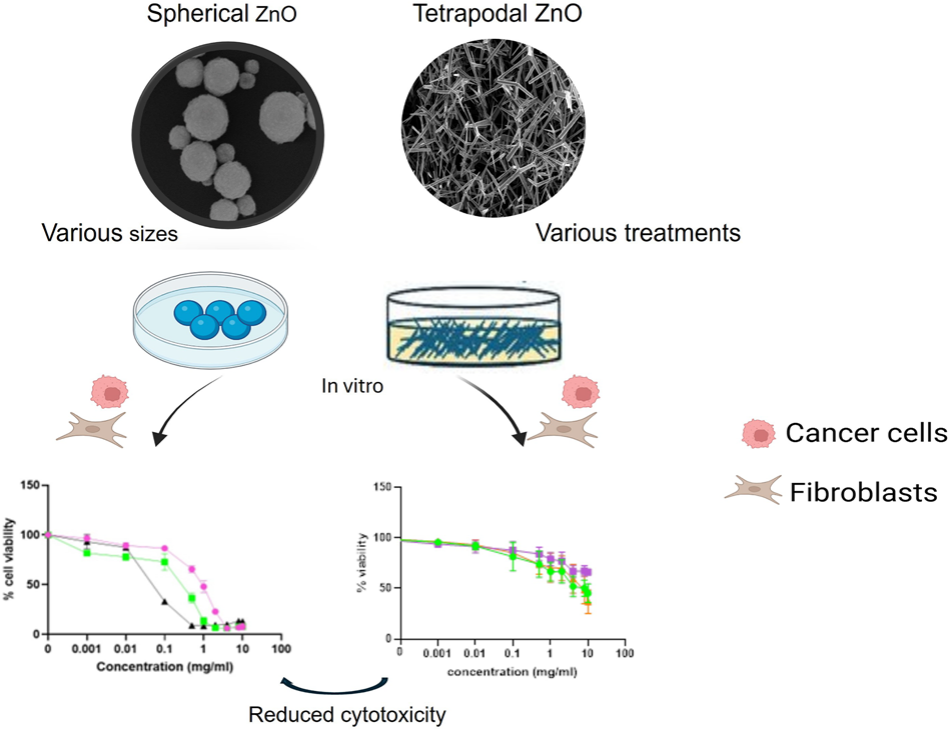

## Introduction

Nanoparticles have garnered significant interest in cancer treatments because of their capacity to target tumor cells specifically while reducing toxicity to healthy tissues. Through improved targeting, decreased systemic side effects, and increased sensitivity, nanotechnology has brought about novel approaches that have improved radiotherapy, chemotherapy, and cancer diagnostics [1, 2]. With their ability to deliver drugs precisely, nanoparticles (NPs) present a promising way to reduce the side effects that are frequently associated to traditional chemotherapeutic agents [2, 3]. Furthermore, NPs composition determines their varied chemical, structural, and biological characteristics, which offer exceptional versatility for a range of biomedical applications [4]. These attributes make NPs highly effective as drug delivery vehicles, allowing for controlled and targeted release of therapeutics [5, 6].

Because of their distinct physicochemical characteristics, biocompatibility, and biodegradability, zinc-based nanoparticles, such as zinc oxide (ZnO) and zinc peroxide (ZnO₂), have drawn considerable attention among the various types of NPs investigated for various cancer cells [7–9]. Notably, pH-responsive stability of ZnO is particularly beneficial; they remain stable at physiological pH (7.4) but dissociate to release Zn^2+^ ions in acidic environments (pH < 6.5), which is typical of the tumor microenvironment [10–15]. This pH-dependent dissolution facilitates the release of Zn²⁺ ions, which may accumulate intracellularly and contribute to cytotoxic responses in cancer cells. [16–25].

In addition to ZnO, ZnO₂ nanoparticles have been investigated for cancer cells elimination using underwater Leidenfrost dynamic chemistry [26]. This method allows for finer control over nanoparticle size and structure by spatially separating nucleation (in an overheated zone) from growth (in a cooler region), in contrast to conventional synthesis methods where nucleation and growth occur simultaneously. Additionally, the nano-micro ZnO tetrapods (T-ZnO) are the subject of this study. Flame transport synthesis (FTS) has recently made their large-scale production possible [27]. Their therapeutic potential is further supported by T-ZnO unique tetrapod morphology, which improves cellular interactions and promotes zinc ion release.

While earlier research has documented T-ZnO and ZnO₂ NPs cytotoxicity in a variety of cell lines (e.g., Jurkat ATCC, PBMC, HT29, L929Ts) [26, 28], there is little information on their effects on breast cancer cells. The ideal NPs concentration and size for successful treatment are also still unknown. The current work fills these gaps by comparing and methodically evaluating the cytotoxic effects of micro-nano T-ZnO and spherical ZnO₂ NPs of various sizes on MCF-7 breast cancer and healthy fibroblast (RMF-EG) cells. Cytotoxicity assessments were performed over a concentration range of 1 µg/mL to 10 mg/mL to identify their effective therapeutic window and ascertain their potential as micro-nanotherapeutics.

## Materials and methods

### Synthesis of ZnO_2_ nanoparticles

Spherical ZnO₂ nanoparticles were synthesized via Leidenfrost nanochemistry, as previously described [26]. Two size distributions were prepared: Type 1 (50–300 nm) and Type 2 (20–80 nm). The average particle size was controlled by adjusting the concentration of the zinc acetate precursor.

### Spherical ZnO nanoparticles

To assess the influence of particle morphology on cytotoxicity, commercial spherical ZnO nanoparticles (average diameter: 50 nm; Sigma Aldrich, CAS No. 8278-2) were used as a reference, as these are commonly employed in cytotoxicity studies.

### Synthesis of ZnO tetrapods

The freestanding tetrapod-shaped ZnO structures (T-ZnO) were synthesized using a flame transport synthesis (FTS) method. Zinc powder (5 µm, GoodFellow, UK) and polyvinyl butyral (PVB, Mowital B 60H, Kuraray GmbH, Europe) were mixed in a 1:2 ratio and heated at 900 °C for 2 h in a box furnace. The resulting snowflake-like material was collected and characterized for morphology and structure.

### Oxygen treatment on T-ZnO

To modulate oxygen vacancy content, T-ZnO was treated in an oxygen-rich environment [28]. For H₂O₂-etched T-ZnO, 3 g of T-ZnO powder was treated with 10% hydrogen peroxide at 100 °C for 15 min, followed by filtration and drying at 80 °C for subsequent use in cell culture experiments.

### Crushed T-ZnO rods

To investigate the effects of geometry and surface area, T-ZnO was mechanically crushed. Intact T-ZnO powder was dispersed in distilled water and subjected to ultrasonication, inducing structural alterations suitable for further study.

### Scanning electron microscopy (SEM)

For SEM analysis (ZEISS ULTRA plus, 7 kV), ZnO and ZnO₂ nanoparticles were dispersed in ethanol, and 100 µL of the suspension was deposited onto silicon wafers. T-ZnO powders were mounted on carbon tape. Particle sizes were analyzed using ImageJ (ij153-win-java8).

### Raman spectroscopy

Micro-Raman spectra were acquired using a WITec Alpha300 RA spectrometer (532 nm Nd-YAG laser, backscattering configuration) to characterize untreated, crushed, and etched T-ZnO, as well as ZnO₂ and ZnO nanoparticles.

### X-ray diffraction (XRD)

XRD patterns were collected using a Seifert 3000 TT diffractometer (Cu Kα, λ = 0.15406 nm, 40 kV, 40 mA) over a 2θ range of 25–80°, at room temperature, on both planar thin films deposited on a glass substrate.

### Transmission electron microscopy (TEM)

Type 1 and Type 2 ZnO₂ nanoparticles were dispersed in butanol, drop-cast onto Cu lacey TEM grids (Plano GmbH), and imaged using a FEI Tecnai F30 STwin G² (300 kV). Bright-field and dark-field images (STEM/HAADF) were acquired. Elemental analysis was performed via EDX (EDAX System). Particle sizes were measured from three images (50,000×–00,000×) using a custom DigitalMicrograph script. For Type 1, 12 nanoparticles were measured; for Type 2, only estimates were provided due to limited isolated nanoparticles.

### Cytotoxicity assessment

Two ZnO₂ nanoparticle variants were selected for cytotoxicity evaluation. The toxicity profiles of T-ZnO particles were compared based on differences in surface charge (O₂-treated) and morphology (crushed), alongside commercial ZnO nanoparticles as a reference. Nanoparticle concentrations ranging from 0.001 to 10 mg/mL were tested for each type.

#### Cell culture of MCF-7 and RMF-EG

Cytotoxicity was evaluated in MCF-7 breast cancer cells and RMF-EG fibroblasts. MCF-7 cells were cultured in RPMI 1640 with 10% FBS and penicillin/streptomycin (100 U/mL and 100 μg/mL). RMF-EG fibroblasts were maintained in DMEM with identical supplements. Cells were incubated at 37 °C, 5% CO₂, and used at passages P15–P25. At 80% confluence, cells were seeded in 96-well plates (17,000 cells/well for MCF-7; 10,000 cells/well for fibroblasts) and allowed to adhere for 24 h. Nanoparticles (0.001–10 mg/mL) were washed, centrifuged (13,000 rpm), dried (40 °C), weighed, and redispersed by ultrasonication in culture medium (stock: 10 mg/mL). Dispersions were sonicated for 30 min for homogeneity, serially diluted, and added to cells for 48 h. Plates were centrifuged (300 g, 5 min) before readout. Cell viability was determined using the CellTiter-Fluor™ assay (Promega, #G6080), following the manufacturer’s protocol. Fluorescence, using an excitation wavelength of 400 nm and emission wavelength of 505 nm, was measured with a microplate reader (Infinite® 200 PRO, TECAN), and viability was calculated as a percentage of control. The upper concentration limit (10 mg/mL) was used to establish a complete dose-response curve and determine IC₅₀ values for materials of varying potency. These concentrations are not intended to be physiologically representative but serve to define comparative cytotoxic thresholds in vitro. The selectivity index (SI) was calculated as:

$${\bf{SI}}={{\bf{IC}}}_{50}\left({\bf{RMF}}-{\bf{EG}}\,{\bf{fibroblasts}}\right)/{{\bf{IC}}}_{50}\left({\bf{MCF}}-{\bf{7}}\,{\bf{cells}}\right)$$

Where SI > 1 indicates preferential cytotoxicity toward cancer cells.

### Zinc ion release studies

#### Zn^+2^ release in different pH media

Zn²⁺ release from ZnO₂ nanoparticles and untreated T-ZnO (1 mg/mL) was quantified after 24 h incubation at 37 °C in phosphate-buffered saline (pH 7.2, 7.4) and acetate buffer (pH 5.5), simulating physiological and endosomal/lysosomal conditions, respectively. After centrifugation, supernatants were analyzed by UV-Vis spectroscopy (Lambda 900, Perkin Elmer) using Zincon (40 µM, Merck, Germany) as a colorimetric reagent (absorbance at 620 nm). Calibration and blank subtraction were performed as described in the Supporting Information (Fig. S1).

#### Intracellular Zn^+2^ release in MCF-7 cells

MCF-7 cells (500,000/well) were incubated with 1 mg/mL ZnO₂ nanoparticles (20–80 nm) for 2 h and 4 h. After incubation, cells were washed, stained with 25 μM Zinquin (30 min, 37 °C), washed again, and harvested by trypsinization. For dead cell quantification, cells were stained with LIVE/DEAD™ Fixable Far-Red (FRD, 1:5000 in PBS, 20 min, 4 °C), washed, and fixed with 1% paraformaldehyde if not stained with FRD. Samples were analyzed by imaging flow cytometry (Image Stream® X Mark II) detecting Zinquin (435–505 nm) and FRD (642–745 nm).

### Statistical analysis

Data was analyzed using GraphPad Prism 9. For two-group comparisons, paired t-tests were used; for multiple groups, one-way ANOVA followed by Dunnett’s multiple comparisons test was applied. Statistical significance was set at *p* ≤ 0.05. All experiments were performed in triplicate (*n* = 3), and results are expressed as mean ± standard deviation.

## Results

### Scanning electron microscopy

**Fig. 1:**
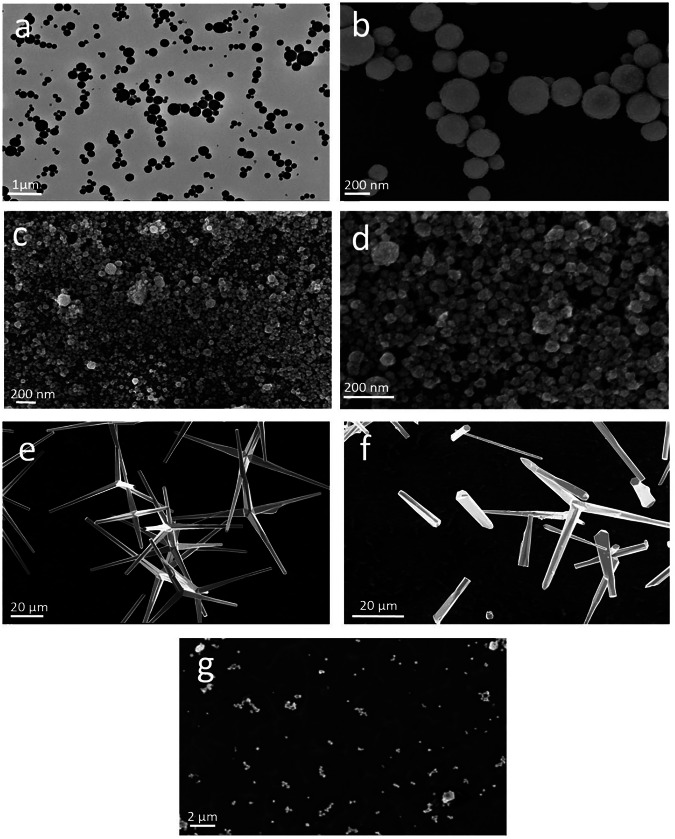
Scanning electron micrograph images of ZnO₂ nanoparticles: **a**, **b** type 1 Nnanoparticles, with average particle sizes ranging from 50 to 300 nm, shown at low (micrometer scale, inverse mode) and high (nanometer scale) magnifications, respectively; **c**, **d** type 2 nanoparticles, exhibiting an average particle size of 20–80 nm at high magnification; **e** untreated T-ZnO particles synthesized via the flame transport method; **f** mechanically crushed T-ZnO particles; and **g** commercial ZnO NPs imaged for comparison

SEM analysis showed distinct morphological differences among the ZnO-based nanomaterials, including ZnO₂ nanoparticles, T-ZnO, and commercial ZnO nanoparticles (Fig.1). Type 1 ZnO₂ nanoparticles appeared as well-dispersed, individual spherical particles with diameters ranging from 50 to 300 nm and showed no significant agglomeration (Fig.1a, b). In contrast, Type 2 ZnO₂ nanoparticles exhibited nearly spherical morphologies with diameters of 20–80 nm, displaying minor aggregation, although discrete nanoparticles remained visible (Fig.1c, d).

SEM images of T-ZnO showed a characteristic 3D architecture composed of four hexagonal cylindrical arms, with base diameters of 1–2 µm, tip diameters of 0.1–0.8 µm, and arm lengths of 15–30 µm. Crushed T-ZnO (Fig.1f) displayed fractured arms, indicating structural changes due to mechanical disruption. Commercial ZnO nanoparticles were observed as small agglomerates and individual spherical particles with an average size of approximately 50 nm, consistent with manufacturer specifications (Fig.1g).

### Raman spectroscopy

**Fig. 2:**
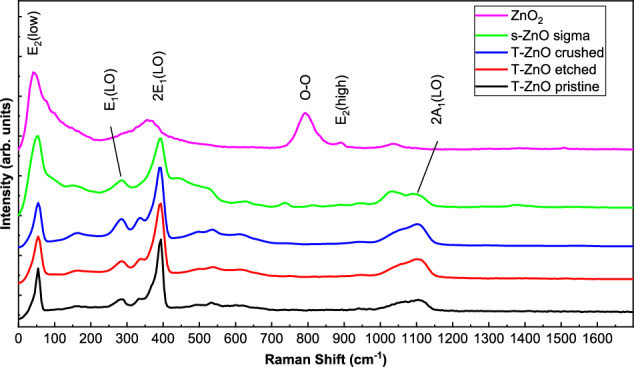
Raman spectra of T-ZnO particles in untreated, etched, and crushed forms, compared with commercially available ZnO nanoparticles (s-ZnO, Sigma-Aldrich) and ZnO₂ nanoparticles

Micro-Raman spectra of T-ZnO (untreated, crushed, etched), commercial ZnO nanoparticles, and ZnO₂ nanoparticles (Fig. 2) displayed well-defined peaks at ~49.5 cm⁻¹ and 389 cm⁻¹, corresponding to the E₂(low) and E₂(high) phonon modes characteristic of the wurtzite ZnO phase [29, 30]. The E₂(low) mode is attributed to Zn sublattice vibrations, while E₂ (high) arises from oxygen vibrations. Additional peaks at 286 and 335 cm⁻¹ were assigned to 2E₂(low), E₂(high)–E₂(low), A₁(TO), and E₁(TO) phonon modes [31, 32]. A broad asymmetric peak at ~1105 cm⁻¹ in all T-ZnO particles was attributed to second-order 2 A₁(LO) and 2E₁(LO) modes [29]. Commercial ZnO exhibited phonon modes at 58.8, 286, and 389 cm⁻¹, with a doublet at 1043 and 1097 cm^-1^.

The ZnO₂ nanoparticles showed five Raman signals at 95, 415, 839, 944, and 1089 cm⁻¹. The peaks at 839 and 944 cm⁻¹ are consistent with nanocrystalline ZnO₂, while a strong O–O stretching mode was observed at 792 cm⁻¹ [33]. Some low-intensity peaks matched those found in other peroxides [34]. Overall, Raman analysis confirmed the successful synthesis and high phase purity of ZnO₂ nanoparticles and T-ZnO particles.

### X-ray diffraction

**Fig. 3:**
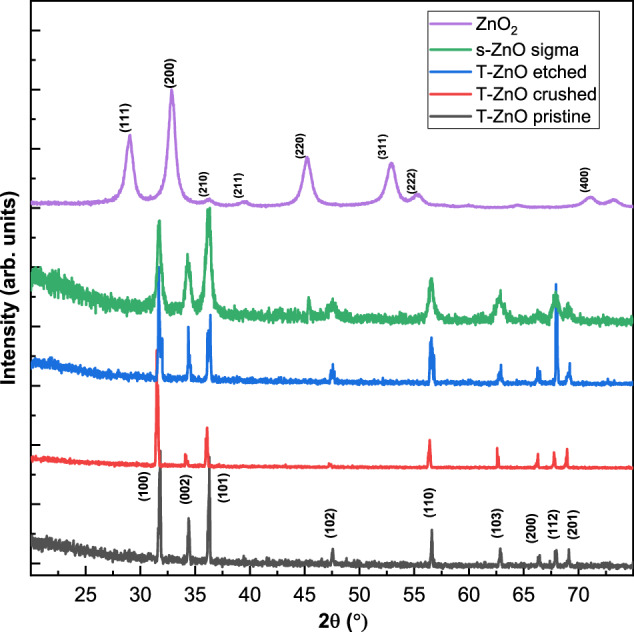
X-ray diffraction (XRD) patterns of T-ZnO particles in untreated, crushed, and etched forms, compared with commercial ZnO nanoparticles (s-ZnO, Sigma Aldrich, CAS No. 8278-2) and ZnO₂ nanoparticles

XRD patterns demonstrated high crystallinity for all ZnO-based materials (Fig. 3). T-ZnO (untreated, etched, and crushed) exhibited sharp diffraction peaks at 31.77°, 34.4°, and 36.2°, corresponding to the (100), (002), and (101) planes of the hexagonal wurtzite ZnO structure (JCPDS 36–1451) [35]. No notable differences were observed among T-ZnO variants. Commercial ZnO nanoparticles demonstrated similar peaks, although with broader profiles, indicative of smaller crystallite sizes. ZnO₂ NPs showed reflections at 2θ = 31.79°, 36.87°, 53.0°, and 63.0°, corresponding to the (111), (200), (220), and (311) planes of cubic ZnO₂ (JCPDS 13-0311), confirming their high crystallinity [36].

### Transmission electron microscopy

**Fig. 4:**
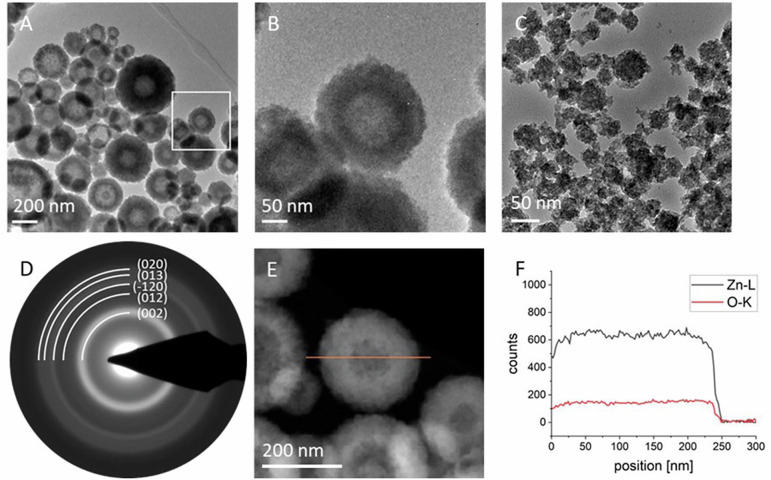
Transmission electron microscopy (TEM) characterization of ZnO₂ nanoparticles. **A**, **B** TEM bright-field images of Type 1 ZnO₂ nanoparticles, with average particle sizes ranging from 50 to 300 nm. **C** TEM bright-field image of Type 2 ZnO₂ nanoparticles, with average particle sizes between 20 and 80 nm. **D** Selected area electron diffraction (SAED) pattern of ZnO₂ nanoparticles (20–80 nm), confirming the presence of the hexagonal ZnO phase. **E** Scanning transmission electron microscopy high-angle annular dark-field (STEM-HAADF) image of ZnO₂ nanoparticles. **F** Corresponding energy-dispersive X-ray (EDX) line scan showing a constant Zn:O ratio across the particle; the 0 nm position indicates the particle edge

TEM analysis of as synthesized ZnO₂ nanoparticles demonstrated spherical nanocrystals, with Type 1 (50–300 nm) particles well-dispersed (Fig. 4a, b) and Type 2 (20–80 nm) nanoparticles partially agglomerated (Fig. 4c). STEM-HAADF and EDX analyses indicated that the particles were hollow, likely due to electron beam effects during TEM imaging [26], and exhibited uniform Zn composition (Fig. 4d). Bright-field TEM images (Fig. 4a, c) confirmed the spherical morphology. Selected area electron diffraction (SAED) patterns showed diffuse concentric rings, indicating nanocrystallinity, with d-spacings of 2.608 Å and 1.913 Å corresponding to the (002) and (012) planes of hexagonal ZnO [37]. This observation contrasts with the XRD results, which indicated a cubic ZnO₂ phase. The discrepancy likely arises from partial beam-induced decomposition of ZnO₂ into ZnO during TEM analysis, as peroxide-based nanomaterials are sensitive to electron irradiation and structural transformation [33]. Evidence of this effect has been reported previously by our group using same ZnO_2_ particles under low-dose, short exposure electron diffraction conditions [26]. Time-resolved electron diffraction (ED) patterns (Fig. S2) initially show sharp rings corresponding to the ZnO_2_ reflection at *d* = 1.756 Å (red mark). However, within few seconds of exposure, the crystallinity changes rapidly, indicating transformation of the particles into hollow spheres composed of ZnO and ZnO_2_ nanoparticles on electron beam irradiation.

### Cytotoxicity assessment

**Fig. 5:**
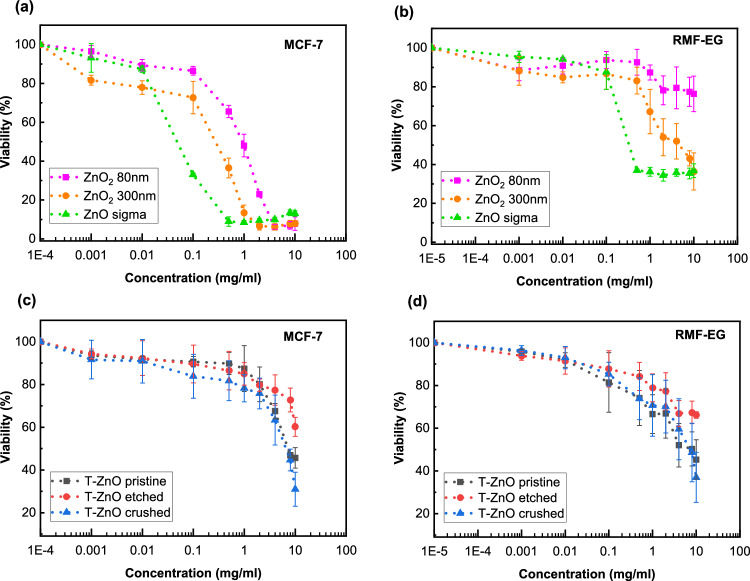
Effect of ZnO₂ nanoparticles and T-ZnO on cell viability, as assessed by the CellTiter-Fluor™ Cell Viability Assay. **a**, **c** Human breast cancer cells (MCF-7) were seeded at 17,000 cells/cm²; **b**, **d** normal fibroblasts (RMF-EG) were seeded at 10,000 cells/cm². Cells were treated with particle concentrations ranging from 0.001 to 10 mg/mL for 48 h. Data represent mean ± SE from three independent experiments (*n* = 3)

Commercial ZnO nanoparticles exhibited an IC₅₀ of 0.042 mg/mL, consistent with literature values for spherical ZnO NPs (0.02–0.05 mg/mL) (Fig. 5a, Table 1) [38]. Both self-synthesized ZnO₂ nanoparticle groups induced cytotoxicity in MCF-7 breast cancer cells, with Type 1 nanoparticles (50–300 nm) showing a lower IC₅₀ (0.182 mg/mL) than Type 2 nanoparticles (20–80 nm, IC₅₀ = 0.73 mg/mL) (Table 1). Notably, commercial ZnO nanoparticles also caused pronounced cytotoxicity in fibroblasts (IC₅₀: 0.167 mg/mL, Fig. 5b), whereas ZnO₂ nanoparticles had minimal effects on fibroblast viability (IC₅₀ = 0.88 mg/mL for Type 1; IC₅₀ not reached for Type 2, even at concentrations up to 10 mg/mL). At 1 mg/mL, Type 2 ZnO₂ nanoparticles reduced fibroblast viability by only 5%, while inducing approximately 40% cell death in MCF-7 breast cancer cells. At 10 mg/mL, Type 2 nanoparticles caused 95% cell death in MCF-7 breast cancer cells but only a slight (~15%) reduction in fibroblast viability. These results indicate that ZnO₂ nanoparticles, particularly Type 2 (20–80 nm), exert cytotoxic effects on malignant cells while sparing non-malignant fibroblasts.

**Table 1:** Effects of nanoparticles and ZnO tetrapods on the half-maximal inhibitory concentration (IC₅₀) values in MCF-7 breast cancer cells and RMF-EG fibroblast cells

	Cell Type	20–80 nm ZnO_2_	50–300 nm ZnO_2_	Commercial ZnO	Untreated T-ZnO	Crushed T-ZnO	Etched T-ZnO
IC_50_ mg/mL	MCF-7	0.730 ± 0.01	0.182 ± 0.01	0.042 ± 0.01	6.404 ± 0.6	6.272 ± 1.4	Not reached
	RMF-EG	Not reached	0.879 ± 0.05	0.167 ± 0.02	7.822 ± 0.5	7.523 ± 1.5	Not reached

The cytotoxicity of synthesized T-ZnO particles, both untreated and following etching or crushing, was evaluated in MCF-7 breast cancer cells and RMF-EG fibroblasts. Cell viability was assessed across a T-ZnO concentration range of 0.001–10 mg/mL (Fig. 5c, d). In MCF-7 breast cancer cells (Fig. 5c), all T-ZnO variants maintained high cell viability at concentrations up to 1.0 mg/mL. However, at concentrations above 1.0 mg/mL, a concentration-dependent reduction in viability was observed. Notably, etching or crushing did not significantly affect the cytotoxic response relative to untreated T-ZnO.

IC_50_ values, summarized in Table 1, indicate that T-ZnO tetrapods are less cytotoxic than spherical ZnO nanoparticles in both MCF-7 and RMF-EG cells. Among the tetrapodal forms, crushed T-ZnO exhibited the greatest cytotoxicity, with IC_50_ values of 6.27 mg/mL for MCF-7 and 7.52 mg/mL for RMF-EG cells. Crushed T-ZnO induced a more pronounced reduction in MCF-7 cell viability relative to etched or untreated T-ZnO. At the highest tested concentration (10 mg/mL), crushed T-ZnO reduced MCF-7 cell viability to approximately 30%. In contrast, untreated and etched T-ZnO reduced viability by 55% in MCF-7 and 40% in RMF-EG cells at this concentration. In RMF-EG fibroblasts (Fig. 5d), all T-ZnO variants demonstrated cytotoxicity trends like those observed in MCF-7 cells, indicating consistent effects across cell types. The observed IC_50_ values are consistent with previous studies; for example, Papavlassopoulos et al. reported IC_50_ values of 5.3–6.1 mg/mL for T-ZnO [28], while Heng et al. and Ahamed et al. reported significantly lower IC^50^ values (0.02–0.05 mg/mL) for spherical ZnO nanoparticles [38, 39]. Overall, these results demonstrate that T-ZnO exhibits lower cytotoxic potency compared to spherical ZnO and ZnO_2_ nanoparticles in both MCF-7 breast cancer cells and RMF-EG fibroblasts.

To quantitatively evaluate preferential cytotoxicity, the SI was calculated as the ratio of IC₅₀ values in RMF-EG fibroblasts to MCF-7 breast cancer cells. As shown in Table 2, ZnO₂ nanoparticles exhibited higher SI values compared to other materials, with the 20–80 nm (Type 2) nanoparticles showing the greatest selectivity (SI > 10), followed by the 50–300 nm (Type 1) with a SI of 4.8. In contrast, commercial ZnO nanoparticles showed moderate selectivity (SI ≈ 4.0), while tetrapodal ZnO displayed minimal selectivity (SI ≈ 1.2), indicating comparable cytotoxic effects in both cell types.

**Table 2:** Degree of in vitro selectivity independent of the test concentration

Material	IC₅₀ MCF-7 (mg/mL)	IC₅₀ RMF-EG (mg/mL)	Selectivity Index (SI)
ZnO₂ (20–80 nm)	0.73	>10 (not reached)	>13.7
ZnO₂ (50–300 nm)	0.182	0.879	4.83
Commercial ZnO	0.042	0.167	3.98
T-ZnO (crushed)	6.27	7.52	1.20
T-ZnO (untreated)	6.40	7.82	1.22

To examine dose relevance, cytotoxicity profiles were further analysed within a physiologically relevant concentration range (0.01–1 mg/mL), summarized in Table 3. In this range, ZnO₂ nanoparticles (20–80 nm) induced a progressive decrease in MCF-7 cell viability, whereas RMF-EG fibroblasts maintained high viability, yielding an increasing differential effect (~15–35%) with increasing concentration. ZnO₂ nanoparticles (50–300 nm) exhibited a similar but less pronounced trend, with moderate MCF-7 viability loss and a smaller decline in fibroblast viability. Conversely, commercial ZnO nanoparticles reduced viability in both MCF cell types to a similar extent across the same concentration range, indicating limited selectivity. T-ZnO, including crushed variants, produced minimal cytotoxic effects in both cell types, with no distinct differential response. Collectively, these results demonstrate that in vitro cytotoxicity remained within a biologically relevant concentration range.

**Table 3:** Cytotoxic effects of ZnO-based materials in MCF-7 breast cancer cells and RMF-EG fibroblasts across the biologically relevant concentration range 0.01–1 mg/mL

Material	Dose (mg/mL)	MCF-7 Viability (%)	RMF-EG Viability (%)	Δ Viability (Selectivity)
ZnO₂ (20–80 nm)	0.01	~90–95	~95–100	Minimal
	0.10	~75–80	~90–95	~15–20%
	1.00	60	~95	**~35%**
ZnO₂ (50–300 nm)	0.01	~85–90	~95–100	Minimal
	0.10	~65–75	~85–90	~15–20%
	1.00	~50–60	~80–85	~20–30%
Commercial ZnO	0.01	~70–80	~80–90	Low
	0.10	~40–50	~50–60	Low
	1.00	~20–30	~30–40	Low
T-ZnO (all types)	0.01	~95–100	~95–100	None
	0.10	~90–95	~90–95	None
	1.00	~85–90	~85–90	None

Values are derived from dose–response profiles. Δ Viability denotes the intercellular difference and serves as a proxy for in vitro selectivity

### Zinc ion release

#### Zn^+2^ ions release in different pH media

**Fig. 6:**
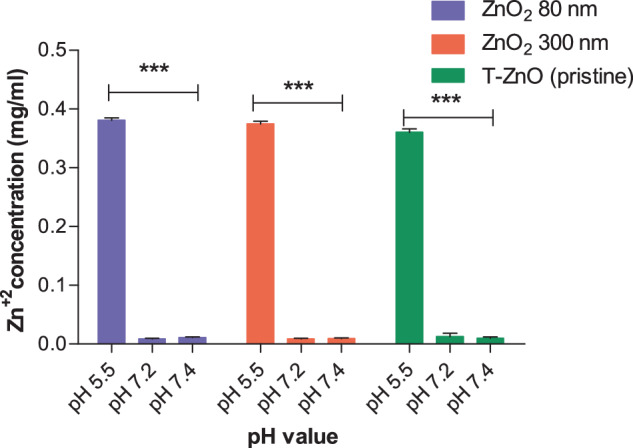
Zn²⁺ ion release from various ZnO_2_ nanostructures and T-ZnO in phosphate-buffered solutions and cell culture medium at pH 5.5, 7.4, and 7.2. ZnO₂ nanoparticles (20–80 nm and 50–300 nm) and tetrapodal ZnO (T-ZnO) exhibited significantly higher Zn²⁺ release at acidic pH 5.5 compared to physiological pH conditions (PBS, pH 7.4; cell culture medium, pH 7.2). Data are presented as mean ± SD (*n* = 3). *p < 0.001 indicates statistically significant differences compared to other pH values

ZnO₂ nanoparticles and T-ZnO particles were dispersed in PBS (pH 7.4) and cell culture medium (pH 7.2) to assess Zn²⁺ release under physiological conditions, and in acetate buffer (pH 5.5) to mimic the endolysosomal environment (Fig. 6). At pH 5.5, both ZnO₂ NPs and T-ZnO released 360–380 µg/mL of Zn²⁺ ions, with no significant differences between particle types. In contrast, Zn²⁺ release was minimal in neutral pH media (9–11 µg/mL after 24 h). These results indicate a pronounced pH-dependent release profile, with particle size and morphology exerting negligible influence under the bulk dissolution conditions.

#### ZnO_2_ particles uptake

**Fig. 7:**
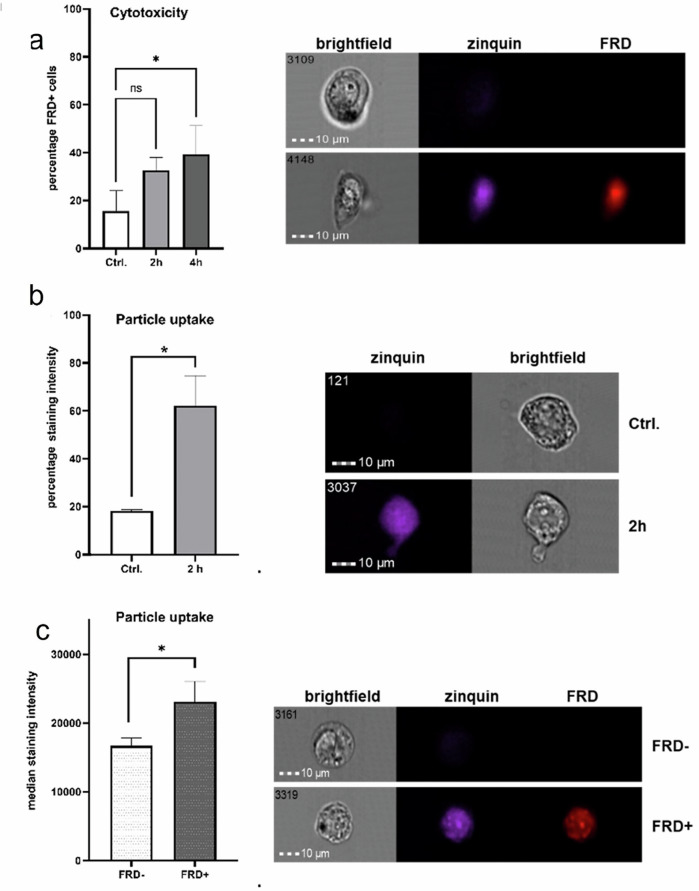
**a** Percentage of FRD-positive (FRD⁺) dead cells following 2 and 4 h of ZnO₂ nanoparticle treatment. **b** Cellular uptake of ZnO₂ nanoparticles assessed by Zinquin fluorescence intensity in untreated cells and cells incubated with ZnO₂ nanoparticles for 2 h. **c** Comparative uptake of ZnO₂ nanoparticles, indicated by Zinquin fluorescence intensity, in viable (FRD⁻) versus dead (FRD⁺) cells after 2 h of ZnO₂ nanoparticle exposure. For panels (**a**–**c**), representative fluorescence images are shown: Zinquin staining (purple) marks ZnO₂ nanoparticle uptake, while FRD staining (red) identifies dead cells. Untreated controls are included for comparison. Scale bars: 10 µm. * Denotes a *p* value < 0.05

To investigate the relationship between ZnO₂ nanoparticle uptake and cytotoxicity in MCF-7 breast cancer cells, quantitative imaging cytometry was performed following 2 h and 4 h incubations. Cell death was visualized by FRD staining, and Zn²⁺ uptake was monitored using the Zinquin fluorescent probe. Apoptosis was evident after 2 h (~30%) and increased after 4 h (>40%), accompanied by decreased Zinquin fluorescence due to Zn²⁺ efflux following loss of membrane integrity (Fig. 7a). Therefore, subsequent analyses focused on the 2 h time point. ZnO₂-treated cells exhibited over threefold higher fluorescence intensity compared to controls, indicating efficient particle uptake (~60%) and intracellular dissolution. Representative images showed diffuse intracellular particle signals (Fig.7b). Quantitative analysis showed a significantly higher proportion of double-stained dead cells (58.07%) compared to living cells (41.9%), supporting the conclusion that ZnO₂ NPs are internalized by cancer cells and induce cell death through intracellular Zn²⁺ accumulation and particle dissolution (Fig.7c). However, the lower proportion of double-stained dead cells in RMF-EG (23.6%; Fig. S3) compared to MCF-7 indicates that although ZnO₂ nanoparticles were internalized by fibroblasts cells, the resulting Zn²⁺ accumulation did not cause substantial death (Fig. S3).

## Discussion

This study demonstrates that ZnO₂ nanoparticles exhibited differential cytotoxic effects in vitro, showing higher cytotoxicity toward MCF-7 breast cancer cells than RMF-EG fibroblasts. In contrast, commercial ZnO nanoparticles reduced viability in both cell types, whereas ZnO₂ nanoparticles, especially the 20–80 nm fraction, produced comparatively lower effects in fibroblasts under the same in vitro experimental conditions.

The anticancer potential of ZnO and ZnO₂ NPs has been investigated in the past, primarily through in vitro experiments, using cancer cell lines like MCF-7, MDA-MB-231, and HT-29 [40, 41]. However, a significant drawback has been the high cytotoxicity observed in non-malignant cells [42]. The present findings indicate improved in selectivity under in vitro conditions. Earlier reports suggest that ZnO-based nanoparticles can induce cytotoxicity via reactive oxygen species (ROS) generation, oxidative stress, and apoptosis [23, 43]. Although these mechanisms may account for the observed effects, they were not directly investigated in this study and therefore cannot be confirmed within this experimental model.

Our findings stand out by demonstrating that ZnO₂ NPs demonstrated a lower toxicity to RMF-EG fibroblasts while retaining potent cytotoxicity against MCF-7 breast cancer cells. A key strength of this study is the selection of RMF-EG fibroblasts as a non-malignant control. Although fibroblasts, epithelial, and endothelial cells are among the non-malignant cell types that have been used in earlier studies, the particular use of RMF-EG fibroblasts is novel. In contrast, T-ZnO showed weaker and less selective cytotoxicity across both two cell types. Minor modifications such as mechanical crushing or oxygen treatment slightly affected cytotoxicity, likely due to changes in particle morphology and subsequent cell-particle interactions [44, 45]. Further investigation is required to elucidate the underlying structure-function relationships.

Comparative cytotoxicity assays demonstrated that spherical ZnO₂ and ZnO nanoparticles were more cytotoxic to MCF-7 breast cancer cells than T-ZnO, likely due to their enhanced cellular uptake via endocytosis. Once internalised, these nanoparticles dissolve within acidic endolysosomal compartments, releasing Zn²⁺ ions into the cytosol and triggering cell death [21, 22]. Importantly, the surfactant-free ZnO₂ NPs synthesized in this study demonstrated superior stability, reduced aggregation, and minimal environmental impact compared to conventionally produced counterparts, as they do not require harmful surfactants. In contrast, the larger, tetrapodal morphology of T-ZnO limits its cellular internalization, precluding direct activation of intracellular cell death pathways. Instead, T-ZnO-induced cytotoxicity is likely attributable to non-specific membrane damage caused by the sharp tetrapod arms. Although Zn²⁺ release was comparable for ZnO₂ and T-ZnO under acidic conditions, their cytotoxic profiles differed, indicating that dissolution alone did not govern cytotoxicity. Instead, differences in cellular uptake and particle-cell interactions likely played a more significant role.

Overall, the results demonstrate that particle size, morphology, and cellular uptake influenced in vitro cytotoxic responses, with ZnO₂ nanoparticles showing preferential effects in MCF-7 cells compared to RMF-EG fibroblasts under the tested in vitro conditions.

## Conclusions

The cytotoxicity of various micro- and nano-structured ZnO and ZnO_2_ materials was effectively assessed through comprehensive experimentation. This study has elucidated that these materials, when exposed to MCF-7 breast cancer cells, can induce dose-dependent cytotoxic effects in vitro. Specifically, ZnO_2_ nanoparticles exhibited increased cytotoxicity towards MCF-7 breast cancer cells in comparison to fibroblasts. Moreover, alterations in T-ZnO properties induced by oxygen treatment or crushing resulted in slight modifications in their cytotoxic behavior. Importantly, our results demonstrate that both ZnO_2_ and commercially available ZnO nanoparticles (spherical) applied herein exerted greater toxicity towards MCF-7 breast cancer cells when compared to tetrapodal-shaped T-ZnO. Cellular uptake experiments further underscored the potential of ZnO_2_ nanoparticles as platforms capable of intracellularly releasing zinc ions, thereby inducing cell death. Overall, the observed differences in cytotoxicity of ZnO₂ nanoparticles toward MCF-7 and RMF-EG fibroblasts likely reflect variations in cellular uptake and intracellular Zn²⁺ release. These findings are limited to two cell lines, and additional studies are needed to validate reproducibility across other cell models and complex biological systems.

## Supplementary information

Supplementary Information

## Data Availability

The data that support the findings of this study are available from the corresponding authors upon request.
