# Supplementary material for: Selective cytotoxicity of zinc peroxide and tetrapodal zinc oxide micro-nanoparticles against breast cancer cells: synthesis, characterization, and therapeutic potential
Source: J Mater Sci Mater Med. 2026 Jun 24;37(1):82. doi: 10.1007/s10856-026-07091-6 (PMC13294158; doi:10.1007/s10856-026-07091-6)
Supplement: Supplementary file 1 — Supplementary Information [file 10856_2026_7091_MOESM1_ESM.docx]

Supporting Information for

**Selective Cytotoxicity of Zinc Peroxide and Tetrapodal Zinc Oxide Micro-Nanoparticles Against Breast Cancer Cells: Synthesis, Characterization, and Therapeutic Potential**

*Tehseen Riaz^*1,5^, Marcus Lettau^2^, Mady Elbahri^3^, Marie Elis^4^, Nicholas Dunne^5-12^, Tanya J. Levingstone^5^, Jörg P. Weimer^13^, Marion van Mackelenbergh^13^, Norbert Arnold ^13^, Nicolai Maass^13^, Dirk Bauerschlag^14^, Fabian Schütt^1^, Rainer Adelung^1^, Nina Hedemann^13,15^*

^1^Chair for Functional Nanomaterials, Institute for Materials Science, Kiel University, Kaiserstraße 2, 24143 Kiel, Germany

^2^ Institute of Immunology, Universitätsklinikum Schleswig-Holstein (UKSH), Arnold-Heller-Straße 3, 24105, Kiel, Germany

^3^ Department of Chemistry and Materials Science, Aalto University, Finland

^4^ Chair for Synthesis and Real Structure, Institute for Materials Science, Kiel University, Kaiserstraße 2, 24143 Kiel, Germany

^5^ School of Mechanical and Manufacturing Engineering, Dublin City University, D09 NA55, Dublin, Ireland

^6^ Centre for Medical Engineering Research, Dublin City University, D09 NA55, Dublin, Ireland

^7^ Advanced Manufacturing Research Centre (I-Form), School of Mechanical and Manufacturing Engineering, Dublin City University, D09 NA55, Dublin, Ireland

^8^ School of Pharmacy, Queen's University Belfast, 97 Lisburn Rd, Belfast BT9 7BL, United Kingdom

^9^ Biodesign Europe, Dublin City University, D09 NA55, Dublin, Ireland

^10^ Advanced Processing Technology Research Centre, Dublin City University, D09 NA55, Dublin, Ireland

^11^ Research Centre for Medical Devices (CÚRAM), Biomedical Sciences, University of Galway, H91 W2TY, Galway, Ireland

^12^ Advanced Materials and Bioengineering Research Centre (AMBER), Trinity College Dublin, D02 PN40 Dublin, Ireland

^13^ Department of Gynecology and Obstetrics, Universitätsklinikum Schleswig-Holstein (UKSH), Arnold-Heller-Straße 3, 24105, Kiel, Germany

^14^ Clinic and Polyclinic for Gynaecology and Reproductive Medicine, University Hospital Jena, Jena, Germany

^15^ Unit for 3D-Patient Avatars and Personalized Medicine, Department of Gynecology and Obstetrics, Christian-Albrechts-University Kiel &amp; University Hospital Schleswig-Holstein, Kiel, Germany

**Corresponding authors: *Tehseen Riaz (***[***Tehseen.riaz804@gmail.com***](mailto:Tehseen.riaz804@gmail.com)***)***

***)***


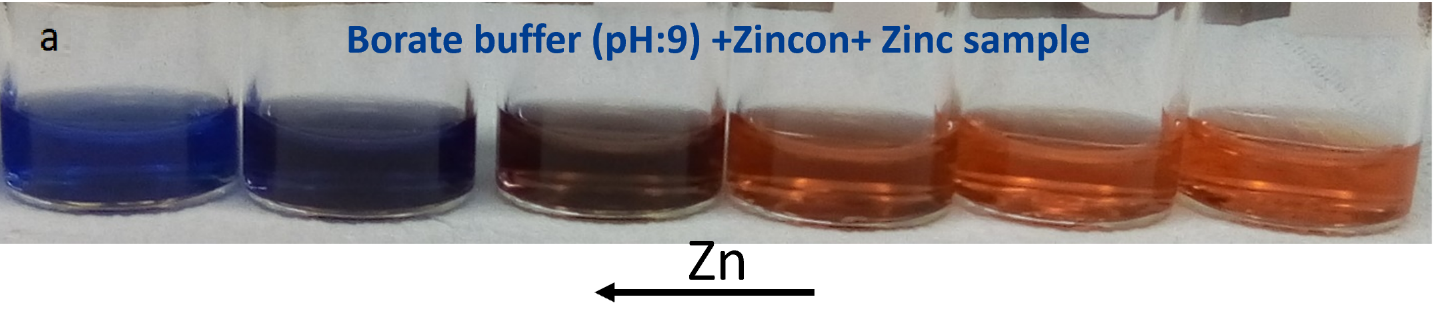


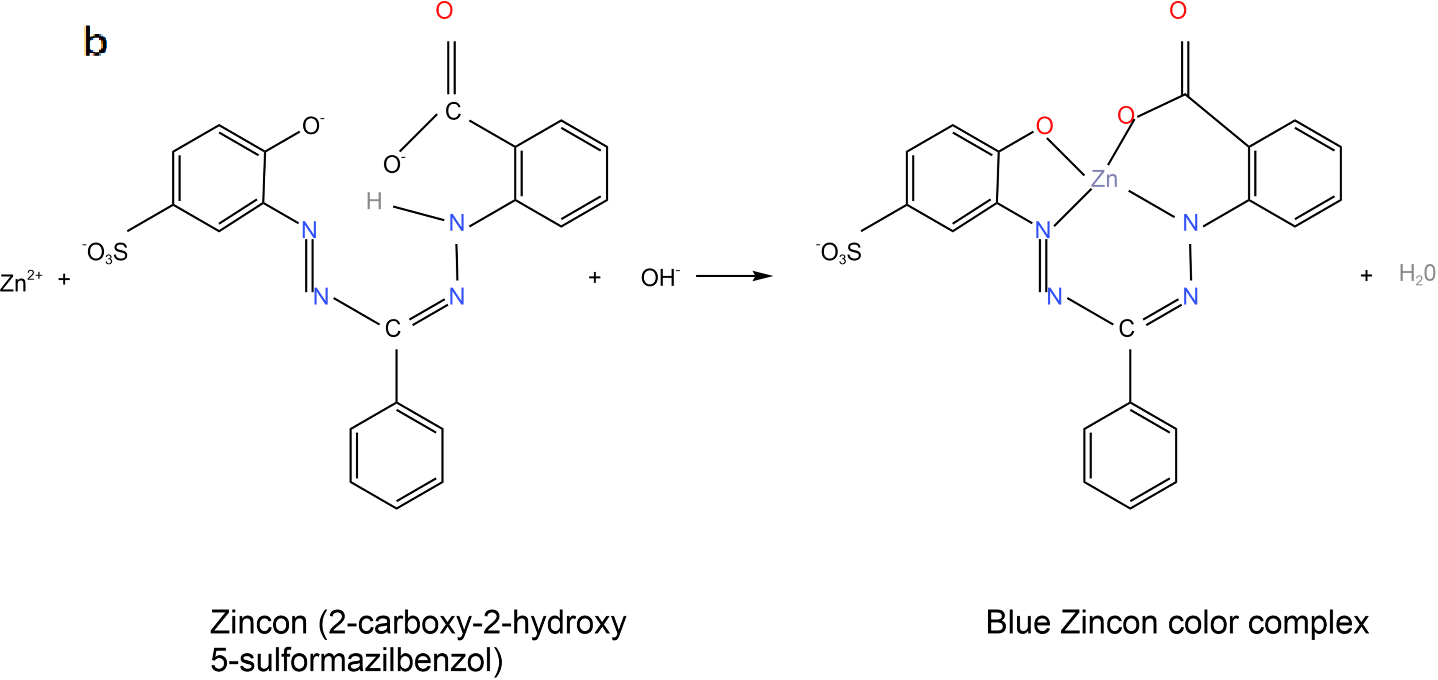


**Fig S1. (a) Preparation of calibration standards for zinc ion detection:** Calibration samples (1 mL total volume) were prepared by adding 25 µL of zinc sulfate stock solutions (ZnSO₄·7H₂O; 10–1000 µg/mL) to 950 µL of borax buffer (50 mM, pH 9.0). Reagent blanks were prepared in parallel by substituting the zinc stock solution with distilled water. Zinc ion complexation was initiated by the addition of 25 µL zincon stock solution (40 µM). Upon addition of zincon, the solution color shifted from red to blue with increasing zinc concentration, enabling colorimetric quantification. **(b) Zincon-zinc complex formation:** Schematic representation of the chemical structures of zincon in its free state and when bound to zinc ions, illustrating the basis for the observed color change.


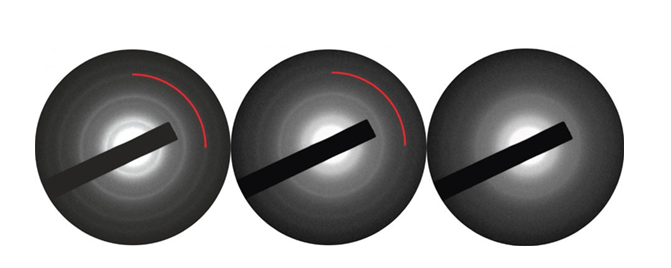


**Fig S2*.***  Time-resolved ED data under low-dose illumination (∼1 s illumination time for each image; the marks are attributed to a reflection at d=1.756 Å being characteristic for ZnO_2_


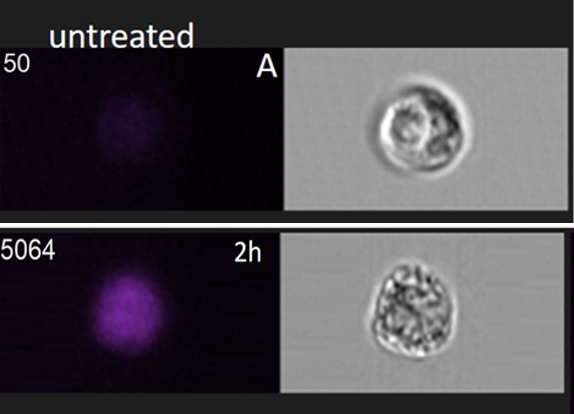


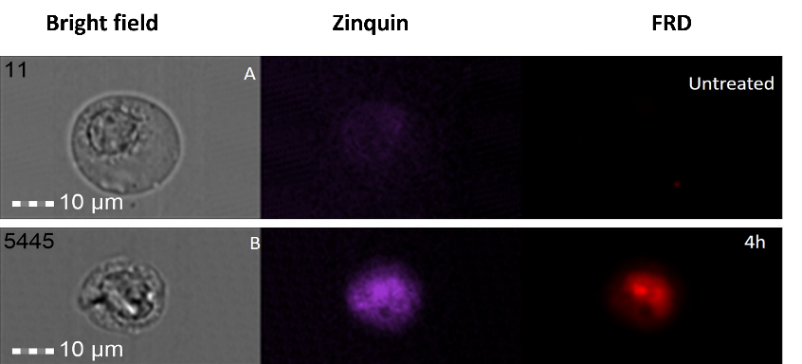


**Fig S3.**  (a) Cellular uptake of ZnO₂ nanoparticles assessed by Zinquin fluorescence intensity in untreated RMF-EG cells and cells incubated with ZnO₂ nanoparticles for 2 h. (b) Percentage of FRD-positive (FRD⁺) dead cells following 2 and 4 h of ZnO₂ nanoparticle treatment. For panels (a–b), representative fluorescence images are shown: Zinquin staining (purple) marks ZnO₂ nanoparticle uptake, while FRD staining (red) identifies dead cells. Untreated controls are included for comparison. Scale bars: 10 µm. * denotes a p-value < 0.05


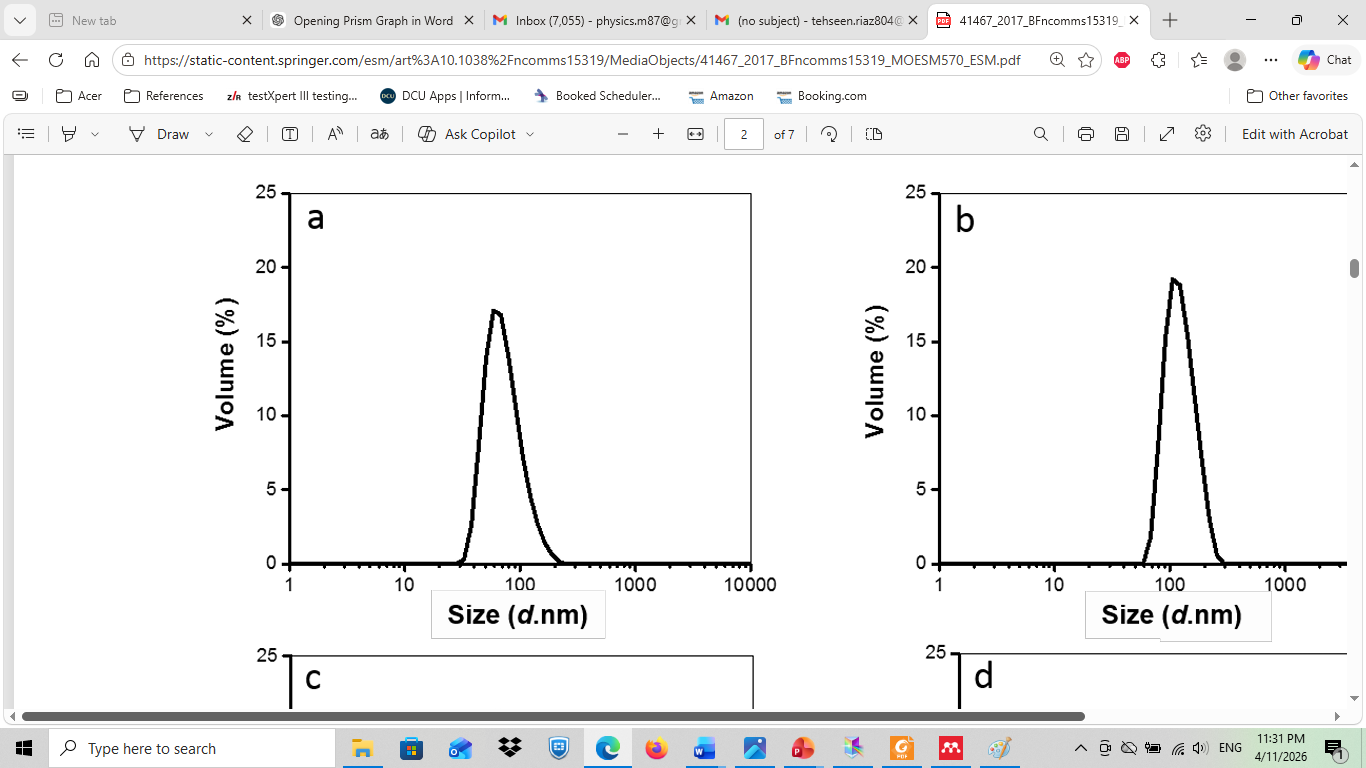


**Fig S4*.***  Particle size distributions of ZnO_2_ nanoparticles with the average size of a) 70 nm (poly dispersity index (PDI)=0.095
